# Impact of *NPSR1* gene variation on the neural correlates of phasic and sustained fear in spider phobia—an imaging genetics and independent replication approach

**DOI:** 10.1093/scan/nsae054

**Published:** 2024-08-21

**Authors:** Elisabeth J Leehr, Leonie S Brede, Joscha Böhnlein, Kati Roesmann, Bettina Gathmann, Martin J Herrmann, Markus Junghöfer, Hanna Schwarzmeier, Fabian R Seeger, Niklas Siminski, Thomas Straube, Anna Luisa Klahn, Heike Weber, Miriam A Schiele, Katharina Domschke, Ulrike Lueken, Udo Dannlowski

**Affiliations:** Institute for Translational Psychiatry, University of Münster, Münster 48149, Germany; Institute for Translational Psychiatry, University of Münster, Münster 48149, Germany; Institute for Translational Psychiatry, University of Münster, Münster 48149, Germany; Institute for Clinical Psychology, University of Siegen, Siegen 57072, Germany; Institute for Biomagnetism and Biosignalanalysis, University of Münster, Münster 48149, Germany; Institute for Psychology, Unit for Clinical Psychology and Psychotherapy in Childhood and Adolescence, University of Osnabrück 49076, Germany; Institute of Medical Psychology and Systems Neuroscience, University of Münster, Münster 48149, Germany; Department of Psychiatry, Psychosomatics, and Psychotherapy, Center of Mental Health, University Hospital of Würzburg, Wurzburg 97080, Germany; Institute for Biomagnetism and Biosignalanalysis, University of Münster, Münster 48149, Germany; Otto-Creutzfeld Center for Cognitive and Behavioral Neuroscience, University of Münster, Münster 48149, Germany; Department of Psychiatry, Psychosomatics, and Psychotherapy, Center of Mental Health, University Hospital of Würzburg, Wurzburg 97080, Germany; Department of Psychiatry, Psychosomatics, and Psychotherapy, Center of Mental Health, University Hospital of Würzburg, Wurzburg 97080, Germany; Department of General Psychiatry, Centre for Psychosocial Medicine, University of Heidelberg, Heidelberg 69115, Germany; Department of Psychiatry, Psychosomatics, and Psychotherapy, Center of Mental Health, University Hospital of Würzburg, Wurzburg 97080, Germany; Institute of Medical Psychology and Systems Neuroscience, University of Münster, Münster 48149, Germany; Department of Psychiatry and Neurochemistry, University of Gothenburg, Gothenburg 41345, Sweden; Department of Psychiatry, Psychosomatics, and Psychotherapy, Center of Mental Health, University Hospital of Würzburg, Wurzburg 97080, Germany; Department of Psychiatry and Psychotherapy, Medical Center-University of Freiburg, Faculty of Medicine, University of Freiburg, Freiburg 79104, Germany; Department of Psychiatry and Psychotherapy, Medical Center-University of Freiburg, Faculty of Medicine, University of Freiburg, Freiburg 79104, Germany; Department of Psychiatry, Psychosomatics, and Psychotherapy, Center of Mental Health, University Hospital of Würzburg, Wurzburg 97080, Germany; Department of Psychology, Humboldt-Universität zu Berlin, Berlin 12489, Germany; German Center for Mental Health (DZPG), partner site Berlin-Potsdam; Institute for Translational Psychiatry, University of Münster, Münster 48149, Germany

**Keywords:** *NPSR1*, imaging genetics, phasic fear, sustained fear

## Abstract

The functional neuropeptide S receptor 1 (*NPSR1*) gene A/T variant (rs324981) is associated with fear processing. We investigated the impact of *NPSR1* genotype on fear processing and on symptom reduction following treatment in individuals with spider phobia. A replication approach was applied [discovery sample: Münster (MS) *n*_MS_ = 104; replication sample Würzburg (WZ) *n*_WZ_ = 81]. Participants were genotyped for *NPSR1* rs324981 [T-allele carriers (risk) versus AA homozygotes (no-risk)]. A sustained and phasic fear paradigm was applied during functional magnetic resonance imaging. A one-session virtual reality exposure treatment was conducted. Change of symptom severity from pre to post treatment and within session fear reduction were assessed. T-allele carriers in the discovery sample displayed lower anterior cingulate cortex (ACC) activation compared to AA homozygotes independent of condition. For sustained fear, this effect was replicated within a small cluster and medium effect size. No association with symptom reduction was found. Within-session fear reduction was negatively associated with ACC activation in T-allele carriers in the discovery sample. *NPSR1* rs324981 genotype might be associated with fear processing in the ACC in spider phobia. Interpretation as potential risk-increasing function of the *NPSR1* rs324981 T-allele via impaired top-down control of limbic structures remains speculative. Potential association with symptom reduction warrants further research.

## Introduction

Specific phobias are among the most frequent mental disorders with a 12-month prevalence of ∼6.4% (Wittchen et al. 2011, Kessler et al. 2012). The most common form of specific phobias is the subtype of spider phobia (Bandelow and Michaelis 2015). Even though exposure therapy is highly effective (Wolitzky-Taylor et al. 2008, Wechsler et al. 2019) and recommended as the first-line treatment (Bandelow et al. 2021), about one-third of patients do no benefit significantly (Choy et al. 2007, Loerinc et al. 2015). Therefore, a better understanding of their neurobiological and molecular genetic basis and variance in treatment outcome may advance treatment and boost treatment response by identifying particularly vulnerable patient subgroups.

The heritability of animal phobia is moderate with ∼30% (Van Houtem et al. 2013, Eaton et al. 2018). One relevant candidate in the field of genetic risk factors for fear and anxiety is the neuropeptide S receptor 1 (*NPSR1*) gene (Pape et al. 2010). The neuropeptide S (NPS) consists of 20 amino acids and its receptor mRNA is widely expressed in the central nervous system (Xu et al. 2004, Pape et al. 2010). In humans, the T-allele of the functional *NPSR1* A/T (Asn107Ile) single-nucleotide variant (rs324981) leads to an increased expression of the receptor and to a ten-fold efficacy of NPS at the receptor (Reinscheid et al. 2005, Bernier et al. 2006). The more active *NPSR1* T-allele has been associated with panic disorder in women, elevated anxiety sensitivity and increased heart rate together with higher symptom report (Domschke et al. 2011) and in interaction with life events with increased anxiety levels (Klauke et al. 2014, Schiele et al. 2020). A meta-analysis of different candidate genes found *NPSR1* variant (rs324981) to be nominally associated with panic disorder (Howe et al. 2016). Also, increased fear ratings in a Pavlovian conditioning paradigm (Raczka et al. 2010), subjectively higher stress levels to stress anticipation, and higher cortisol levels while facing acute psychosocial stress (Kumsta et al. 2013) were linked with the T-allele. Even though spider phobia is a prototypical anxiety disorder, there is a research gap regarding its association with *NPSR1* variants.

A basic neural model of anxiety disorders suggests limbic hyperactivation elicited by, i.e. threat with potentially impaired regulation from prefrontal regions (Etkin et al. 2015). Further, there is evidence for negative connectivity between limbic and prefrontal regions in anxiety disorders (Xu et al. 2019). Building on this, imaging genetic studies, both healthy individuals (Dannlowski et al. 2011, Streit et al. 2014) and patients with panic disorder (Gechter et al. 2019) carrying the T-allele showed heightened amygdala activity during threat confrontation. Patients with panic disorder carrying the T-allele showed significantly decreased activity in the anterior cingulate cortex (ACC) during the processing of fearful faces, whereas the AA genotype could be associated with increased activation in the dorsolateral prefrontal cortex (dlPFC) and the lateral orbitofrontal cortex (OFC) (Domschke et al. 2011). In functional near-infrared spectroscopy studies, increased activation in the medial prefrontal cortex (mPFC) and in the dlPFC to fear-relevant stimuli was observed in AA homozygotes (Tupak et al. 2013), whereas in another study T-allele carriers showed a signal increase to negative pictures in these regions (Guhn et al. 2015). In healthy adolescent TT homozygotes, a reduction in fronto-limbic connectivity was discerned (Domschke et al. 2017). Likewise, patients with panic disorder carrying the T-allele showed the highest activation in the inferior OFC and healthy controls with the AA genotype the lowest during symptom provocation through agoraphobia-related pictures (Gechter et al. 2019). Results regarding specific phobia are yet missing.

To better understand the underlying neural networks of phobic fear, it is important to distinguish between the lasting state of anticipating an unpredictable threat, called sustained fear, and the immediate response to an explicit predictable threat cue, called phasic fear. At first glance, phasic fear could be classified as “archetype of a [phasic] ‘fear disorder’” (Grillon 2008, 424) describing the immediate response to a confrontation with the phobic stimulus. However, more severe everyday impairments emerge from sustained fear, e.g. in form of the hypervigilant monitoring of environment, contributing to avoidance behavior (Dubrovsky et al. 1978, Andrews et al. 1994, Öst 1996). Whereas the well-studied neural basis of phasic fear is mainly represented by the amygdala and the insula (Etkin and Wager 2007), knowledge about the neural basis of sustained fear is still inconclusive. Neural correlates of sustained fear in samples with spider phobia have been identified in the anterior cingulate cortex (ACC), insula [associated with anticipatory processes (Simmons et al. 2006)], and bed nucleus of stria terminalis (BNST) (Straube et al. 2007, Münsterkotter et al. 2015). Klumpers et al. (2017) demonstrated that heightened BNST activity, while anticipating a shock, shifts to amygdala activity, when being confronted with the shock, thus corroborating the theory of different interacting neuronal networks for sustained and phasic fear. A differential involvement of the central nucleus of the amygdala and the BNST in threat anticipation and confrontation has been demonstrated in a small sample with patients with spider phobia (Siminski et al. 2021b), and in healthy controls (Siminski et al. 2021a). Furthermore, *NPSR1* gene variation modulated BNST activity in healthy controls, as BNST was more active in healthy T-allele carriers during unpredictable threat anticipation compared to healthy AA homozygotes (Siminski et al. 2021a).

So far, no study has been published that focused on *NPSR1* gene variation and neural correlates of sustained and phasic fear in spider phobia. Furthermore, the association with treatment outcomes has not been addressed as a translational link between basic research and clinical practice. The ACC is a major focus of current research on neural substrates of treatment response in anxiety disorders (Lueken et al. 2016, Picó-Pérez et al. 2022) and a therapy-induced reduction of ACC hyperactivity was shown in individuals suffering from spider phobia (Straube et al. 2006, Goossens et al. 2007).

Based on previous evidence, we defined the amygdala, BNST, insula, and ACC as regions of interest (Münsterkotter et al. 2015). We hypothesized that irrespective of genotype and symptom reduction, phasic fear would be associated with heightened amygdala activity, while the state of sustained fear would be related to activity in the BNST, insula, and ACC. Further, we expected *NPSR1* rs324981 T-allele carriers to show hyperactivity in the amygdala during phasic fear processing compared to AA homozygotes. We expected altered activity in the ACC, BNST and insula during sustained fear processing as a function of *NPSR1* genotype. Finally, we investigated potential associations of imaging genetic effects with symptom reduction in response to virtual reality exposure. All results were analyzed in an independent replication sample.

## Materials and methods

### Study design and sample description

The bicentric study was conducted as part of the Transregional Collaborative Research Center (CRC-TRR58) “Fear, Anxiety, Anxiety Disorders”. The study protocol was approved by the ethics committees of the respective universities (Würzburg: 330/15; Münster: 216-212-b-S) and has been published (Schwarzmeier et al. 2019). The clinical study has been registered at ClinicalTrials.gov (ID: NCT03208400). Taking advantage of the bi-centric data acquisition, analyses were performed separately for each site, using the larger sample from Münster (MS) as discovery sample (*n * = 104) and the sample from Würzburg (WZ) as replication sample (*n* =  81).

Patients had to be diagnosed with specific phobia of the animal subtype (spider phobia) according to the DSM-IV criteria and had to reach at least a sum score of 20 in the Spider Phobia Questionnaire (Klorman et al. 1974). They were aged between 18 and 65 years, right-handed, fluent in German language, and of Caucasian descent (for detailed exclusion criteria see Supplement 1).

We here present post-hoc exploratory analyses supplementing the main research question (Leehr et al. 2021, Chavanne et al. 2023). See Supplement 1 and 2 for detailed description of sample constitution and potential overlaps with other analyses. Based on *NPSR1* rs324981 genotype, the sample was grouped into T-risk-allele carriers (including TT homozygotes and AT heterozygotes) and non-risk-carriers (comprising AA homozygotes) (Domschke et al. 2011).

### Procedure

Recruitment was realized through advertisements, social media, flyers, and posters. The present study included a baseline assessment of clinical and psychometric, behavioral, and (epi-) genetic data. During the *in vivo* behavioral avoidance test (BAT), the patient, sitting 3 m away from a spider in a box, which was placed on a slide, was told to drag the box as close as possible towards him-/herself. At the second visit, structural and functional MRI data were gathered, including the sustained and phasic fear paradigm. Afterwards, patients received a one-session exposure therapy in virtual reality (VRET) and a post-assessment, including the same measurements as at baseline. VRET included a brief psychoeducation on the rationale of behavioral exposure and the exposure to spiders in five different scenarios (Schwarzmeier et al. 2019). Follow-up data (data not presented here) were recorded after 6 months. For detailed information regarding the complete study procedure please see Schwarzmeier et al. (2019).

### Clinical assessments

Diagnoses were ascertained using a structured clinical interview (SCID-I) according to the “Diagnostic and Statistical Manual of Mental Disorders IV” (Wittchen et al. 1997). The Spider Phobia Questionnaire (Klorman et al. 1974) was used to measure symptom severity. A sum score of at least 20 is the cut-off score for clinically significant symptom severity (Hamm, 2006). To assess the level of disgust and fear of spiders, we used the German questionnaire FEAS (Schaller et al. 2006). General symptom-related characteristics were assessed with the State-Trait-Anxiety Inventory (STAI, Spielberger et al. 1970), the Anxiety Sensitivity Index (Taylor et al. 2007), the Intolerance of Uncertainty scale (Buhr and Dugas 2002), and the Beck Depression Inventory (Beck et al. 1996). Percentage of change in symptom severity (SPQ) and behavioral avoidance (BAT) from pre to post as well as within-session fear reduction indexed by the mean change of self-rated fear overall VRET scenarios were assessed.

### Genotyping

A venous EDTA blood sample was taken to genotype for the functional *NPSR1* rs324981 A/T (Asn107Ile) polymorphism according to published protocols (Dannlowski et al. 2011, Domschke et al. 2011, Schiele et al. 2020). First, the DNA isolated from the blood sample was amplified using the primers F: 5´TGCTTTGCATTTCCTCAGTG and R: 5´TTGTCTCATCACATTTGGAAGG. Standard PCR was conducted in a 25 μl volume containing 1 μl genomic DNA, 1 μl of each primer, 0.3 μl Taq DNA polymerase, 1 μl of 2.5 mM dNTPs, 2.5 μl of 15 mM MgCl_2_ and 18.2 μl bidest H_2_O. After a 5 min denaturation at 95°C, 35 cycles were conducted, starting with 45 s at 95°C, followed by 45 s at 58°C and 45 s at 72°C. The PCR was ended with a final extension step of 5 min at 72°C, resulting in an amplicon size of 294 bp. The amplicons were digested with the restriction enzyme *Ase*I for 3 h at 37°C and separated on 2–3% polyacrylamide gel. The products were then visualized with ethidiumbromide under UV light. The T-allele product comprises 294 bp, the A-allele comprises two products, including one with 195 bp and one with 108 bp. Hardy–Weinberg criteria as determined by the online program SNPstats (https://www.snpstats.net/start.htm?) were fulfilled for both samples (discovery sample: *n*_TT_ = 17; *n*_A/T_ = 57; *n*_AA_ = 30; *P = *.32; replication sample: *n*_TT_ = 14; *n*_A/T_ = 43; *n*_AA_ = 24; *P = *.59; combined sample: *n*_TT_ = 31; *n*_A/T_ = 101; *n*_AA_ = 54; *P = *.22) and pass with a minor allele frequency of 0.31 and a genotyping call rate of 100% the quality control.

## fMRI-data: sustained and phasic fear paradigm

The “Sustained and Phasic Fear Paradigm” (SPF), based on the paradigm implemented by Münsterkötter et al. (Münsterkotter et al. 2015), is structured in a block design. There are three active blocks (phasic fear, sustained fear, and no fear), and each is presented five times in a pseudorandomized order. Per active block, 10 images are shown for 1.7 s each, followed by a fixation dot, presented for 300 ms. In the “Phasic Fear” condition, patients are confronted with pictures of spiders. In the “Sustained Fear” condition, participants are told that they might see pictures of spiders and are confronted with pictures of empty rooms and only in the last quarter of the block, in one-third of the runs, spiders are presented. The last condition is the “No fear” condition, in which patients are informed that they will only see empty rooms and are subsequently only confronted with empty rooms. After each active block, an inactive block (baseline) without any stimulus starts, only showing a central dot (15 s), which the participants were asked to fixate. Total duration of the task is 9:45 min.

### Data acquisition and preprocessing

*Data acquisition*. In both Münster (Siemens Prisma) and Würzburg (Siemens Skyra), a 3-Tesla magnetic resonance scanner and homogenized MRI sequences were used. Before the fMRI was conducted, a structural T1 was taken using magnetization-prepared rapid gradient echo (MPRAGE). The T2*-weighted data of the phasic and sustained fear paradigm were collected via an echo planar imaging sequence sensitive to blood oxygenation level-dependent contrast (see Supplement 3 for specific parameters).

*Preprocessing*. An established preprocessing protocol including realignment, unwarp, and normalization to the Montreal Neurological Institute International Consortium for Brain Mapping (MNI) template was performed using SPM 8 (Wellcome Department of Cognitive Neurology SPM8 n.d.). Images were smoothed with a 6-mm full-width at half-maximum Gaussian kernel. See Supplement 3 for details on quality control.

### Statistical analyses

#### Sociodemographic and clinical data

Data analysis was performed using SPSS 28 (IBM 2012). Investigating group differences, χ^2^-tests as well as *t*-tests for independent samples were used.

#### MRI data

##### First-level analysis.

Onsets and durations of the experimental conditions (phasic fear, sustained fear, and no fear) were modeled using the default canonical hemodynamic response function in the context of a General Linear Model in SPM 12. The following two first-level contrasts were used in the subsequent second-level analysis: “phasic > no fear, sustained > no fear.”

##### Second-level analysis.

For all analyses, we applied threshold-free cluster enhancement (Smith and Nichols 2009) with 5000 permutations and family-wise error-corrected significance levels of *P*_FWE_ = .05. To interpret significant *P*-values, we calculated the corresponding Hedge’s *g* and its confidence interval, using a Matlab-script by Gerchen et al. (2021). Please note, that effect size calculation was performed for peak voxel level and that true effect sizes might be somewhat smaller.

General effects of the fear conditions (phasic > no fear and sustained > no fear) were analyzed as proof of expected task activation using a 2 (Group) × 2 (Contrast) ANCOVA with age and sex as covariates of no interest. To perform a region of interest (ROI) analysis, we created one mask including all ROIs; either the ROI definitions according to the Anatomical Automatic Labeling atlas (Tzourio-Mazoyer et al. 2002, Rolls et al. 2020) for the amygdala, the insula, and the ACC or, in the case of the BNST a dilated mask (*x* = −10, *y* = −1, *z* = 2 and *x* = 12, *y* = −2, *z* = 2 with a 4-mm sphere dilated by 1 mm), which has been previously used for this task by Münsterkotter et al. (2015).

The effect of *NPSR1* genotype on correlates of fear processing was analyzed for each fear condition separately. We performed an ANCOVA using the joint mask, including amygdala, insula, ACC, and BNST to analyze the main effect of group (risk versus no risk genotype) and symptom reduction (percentage of change in symptom severity measured by the SPQ), as well as the interaction effect of group and symptom reduction. Age and sex were included in each model as covariates of no interest. We performed a *t*-test for the directed hypothesis concerning the main effect of genotype in the phasic fear condition. For all other hypotheses, we chose *F*-tests. In the case of significant main or interaction effects, Bonferroni-corrected post-hoc tests (*P*-level of .025) were performed. Analyses were repeated in an exploratory whole-brain analysis (results are presented in Supplement 5). Additionally, exploratory correlation analyses between the activation values of significant clusters and change in behavioral avoidance and the within-session fear reduction were performed in SPSS. For effects in the discovery sample, a mask with voxels above the threshold was exported and further used for replication analyses in the replication sample.

## Results

### Sample characteristics

Genotype groups did not differ regarding sociodemographic or clinical data (see Table 1). There was no effect of genotype groups on symptom reduction or change in avoidance behavior after the VRET. Discovery and replication samples did not differ significantly regarding their sample characteristics, except for SPQ pre (WZ > MS; mean sum score difference = 0.52), duration of VRET (WZ > MS; mean duration difference = 8.61 min), and within-session fear reduction (MS > WZ; mean difference = 7.94) (Supplement 4).

**Table 1.** Sample characteristics (mean and s.d.) for *NPSR1* rs324981 low risk (AA) and risk-allele carriers (AT/TT) in the discovery sample.

		AA	AT/TT	Test statistic	*P*-value
**Discovery sample**	N	30	74		
*Sociodemographic variables*					
Sex (f/m)	104	25/5	66/8	0.67	.413
Age (years)	104	27.1 (9.71)	27.43 (7.99)	−0.18	.857
Years of education	103	14.69 (2.58)	14.66 (2.86)	0.05	.964
*Psychological variables*					
SPQ	104	22.439 (1.91)	22.61 (2.02)	−0.41	.685
BAT	104	168.47 (76.85)	163.19 (66.85)	0.35	.728
FEAS Disgust	103	106.93 (15.56)	110.43 (14.79)	−1.07	.286
FEAS Fear	104	101.97 (13.20)	101.60 (10.58)	0.15	.880
STAI-Trait	103	33.55 (6.88)	35 (7.99)	−0.86	.393
ASI-3	103	13.48 (8.96)	14.84 (9.48)	−0.66	.509
UI-18	103	36.90 (10.59)	38.59 (12.30)	−0.65	.514
BDI-II	103	3.28 (3.84)	3.12 (3.04)	0.22	.831
*Intervention related variables*					
Number of scenarios completed	104	4.27 (1.28)	4.42 (1.06)	−0.62	.534
VRET duration (min)	104	78.3 (29.10)	76.84 (23.47)	0.27	.789
SPQ change pre to post (in %)	104	30.96 (14.62)	31.37 (13.22)	−0.14	.889
BAT change pre to post (in %)	104	53.91 (31.46)	47.69 (33.38)	0.87	.384
Within-session fear reduction	104	51.95 (21.68)	55.43 (18.08)	−0.84	.404
**Replication sample**	N	24	57		
*Sociodemographic variables*					
Sex (f/m)	81	20/4	50/7	0.277	.599
Age (years)	81	28.04 (9.26)	28.89 (8.75)	−0.394	.695
Years of education	81	13.54 (3.26)	14.79 (3.28)	−1.566	.121
*Psychological variables*					
SPQ	81	22.58 (2.26)	23.51 (2.45)	−1.586	.117
BAT	81	175.63 (50.36)	166.35 (66.10)	0.615	.540
FEAS Disgust	80	109.21 (10.77)	110.43 (12.71)	−0.411	.682
FEAS Fear	80	99.00 (16.77)	102.63 (12.87)	−1.052	.296
STAI-Trait	81	37.00 (9.55)	35.88 (9.11)	0.499	.619
ASI-3	81	16.21 (11.39)	14.72 (9.40)	0.611	.543
UI-18	81	42.21 (18.05)	39.37 (12.80)	0.803	.424
BDI-II	81	3.88 (4.48)	3.05 (4.16)	0.794	.429
*Intervention related variables*					
Number of scenarios completed	81	4.75 (0.74)	4.65 (1.09)	0.413	.681
VRET duration	81	86.92 (28.40)	85.44 (24.99)	0.233	.816
SPQ change pre to post (in %)	81	30.04 (18.01)	35.60 (15.26)	−1.418	.160
BAT change pre to post (in %)	81	48.74 (21.37)	55.92 (29.04)	−1.092	.278
Within-session fear reduction	81	43.36 (21.36)	48.15 (18.40)	−1.019	.311

Notes: The variable test statistic comprise a Chi^2^-test for group differences regarding the sex distribution and in all other cases T-values. *Value indicate the highest symptom severity; f = female; m = male; SPQ = Spider Phobia Questionnaire (Range 0–31*); BAT = Behavioral Avoidance Test (0–300*); FEAS = Questionnaire assessing fear of spiders (0–126*); STAI-Trait = State-Trait Anxiety Inventory (0–80*); ASI-3 = Anxiety sensitivity index (0–72*); UI-18 = Intolerance of uncertainty (18–90*); BDI-II = Beck’s Depression Inventory-II (0–63*).

### Neural correlates of phasic and sustained fear condition

Irrespective of *NPSR1* rs324981 genotype, phasic fear compared to sustained fear resulted in greater activation not only in the bilateral amygdala, but also in the bilateral insula, and in the bilateral supracallosal ACC (see Table 2). In contrast, sustained fear was associated with greater activation in the bilateral pregenual ACC, predominantly located in the pregenual parts with small extensions into the subgenual and supracallosal ACC, and a small cluster in the left insula (see Table 2). In contrast to our hypothesis, sustained fear was not associated with increased BNST activation.

**Table 2.** Neural correlates of phasic and sustained fear condition (MNI coordinates of peak voxels).

		Discovery sample							Replication sample					
Regions of interest	Side	*k*	*x*	*y*	*z*	*P*_FWE_	ES (95%CI)		*k*	*x*	*y*	*z*	*P*_FWE_	ES (95%CI)
Phasic fear > sustained fear														
Amygdala	R	244	26	−2	−22	.001	1.54 (1.07; 2.01)		77	28	0	−18	.002	1.77 (1.20; 2.34)
	L	202	−24	−2	−24	.001	1.75 (1.28; 2.23)		31	−24	−4	−18	.010	1.75 (1.18; 2.32)
Insula	R	1252	34	0	14	<.001	1.68 (1.21; 2.16)		771	38	8	−10	<.001	1.64 (1.08; 2.21)
	L	1361	−34	0	14	<.001	2.07 (1.59; 2.57)		655	−34	−4	10	<.001	2.09 (1.51; 2.68)
ACC (supracallosal)	R	1	8	6	28	.045	0.69 (0.24; 1.14)		97	2	18	26	.014	1.31 (0.76;1.87)
	L	270	−2	22	26	.007	1.2 (0.66; 1.58)							
Sustained fear> phasic fear														
Insula	R								1	32	22	−4	.050	1.30 (0.75; 1.86)
	L	33	−30	24	−4	.011	1.21 (0.75; 1.68)		23	−30	18	−4	.013	1.57 (1.01; 2.14)
ACC (pregenual and subgenual)	R	656	4	46	22	<.001	1.39 (0.93; 1.87)		428	10	40	16	<.001	1.40 (0.85; 1.97)

Notes: R = right; L = left; ACC = anterior cingulate cortex.

Effects of fear condition could be replicated in the replication sample (see Table 2).

### Association between *NPSR1* genotype and neural correlates of phasic and sustained fear

#### Phasic fear.

We found a main effect of genotype group in an ACC cluster (see Table 3), indicating hypoactivation of the supracallosal ACC during phasic fear processing in T-allele carriers compared to AA homozygotes (ES *g* = 0.76; 90% CI = 0.034–1.18). This effect could not be replicated in the WZ sample (see Table 3). Neither a main effect for symptom reduction, nor an interaction effect of genotype group and symptom reduction could be shown. ACC activation was not correlated significantly with change in behavioral avoidance or within-session fear reduction.

**Table 3.** Association between *NPSR1* genotype and neural correlates of phasic and sustained fear condition (MNI coordinates).

		Discovery sample						Replication sample				
Regions of interest and effects	Side	*k*	*x*	*y*	*z*	*P_FWE_*		*k*	*x*	*y*	*z*	*P_FWE_*
**Phasic fear**												
*T–allele carriers < AA homozygotes*												
ACC supracallosal	L	31	−2	30	−20	.039		–	–	–	–	–
*Symptom reduction*												
		–	–	–	–	–						
Genotype group*symptom reduction												
		–	–	–	–	–						
**Sustained fear**												
T–allele carriers < AA homozygotes												
ACC supracallosal		125	0	32	20	.012						
	R	24	6	16	26	.045						
	L							5	−4	32	24	.038
*Symptom reduction*												
		–	–	–	–	–						
Genotype group*symptom reduction												
		–	–	–	–	–						

Notes: R = right; L = left; ACC = anterior cingulate cortex.

#### Sustained fear.

**Figure 1.**
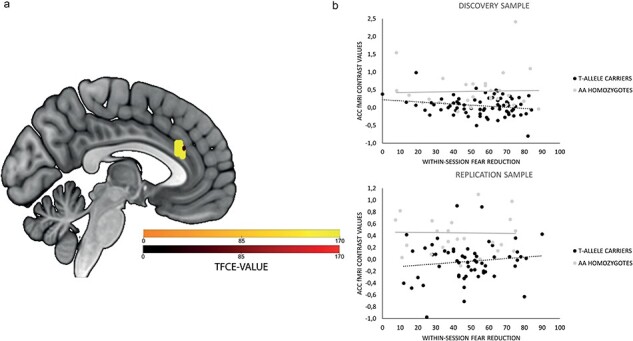
**(A)** Activation differences between *NPSR1* rs324981 T-allele carriers and AA homozygotes in the supracallosal ACC for the contrast sustained versus no fear (T-allele carriers < AA allele homozygotes). **(B)** Correlation between supracallosal ACC activation and within-session fear reduction.

***Note***: **(A)**: Contrast sustained versus no fear (sagittal view: x=-4); results for the discovery sample are displayed in yellow; results for the replication sample are displayed in red. Upper color bar indicates TFCE-values for the discovery sample, Color bar beneath indicates TFC-values for the replication sample. **(B)**: fMRI contrast values were computed by extracting the first eigenvariate of the significant cluster (discovery sample: x=0, y=32, z=20, k=125, pTFCE-FWE=.012, ES g=0.68. 95% CI=0.26-1.1; replication sample: x=-4, y=32, z=24, k=5, pTFCE-FWE=.038, ES g=0.79. 95% CI=0.30-1.29) resulting from the ROI post-hoc analysis *NPSR1* rs324981 T-allele carriers < AA homozygotes. ACC=anterior cingulate cortex.

During sustained fear, T-allele carriers showed a significant hypoactivation in the bilateral supracallosal ACC compared to AA homozygotes (ES *g* = 0.68; 95% CI = 0.26–1.1) (see Table 2 and Fig. 1a). The smaller cluster did not survive *P*-value adjustment after Bonferroni-correction (*P* < .025). The hypoactivation in T-allele carriers could be replicated in the WZ sample in a very small cluster though (ES *g* = .79; 95% CI = 0.30–1.29). No main effects regarding symptom reduction or its interaction with genotype were found. No association between *NPSR1* genotype, insula, and BNST activation could be found.

However, activation in the ACC was negatively correlated with within-session fear reduction in T-allele carriers (*r = *−.239, *P = *.040), whereas in AA homozygotes these variables were not associated (*r = *.031, *P = *.873) (Fig. 1b). No significant correlation between ACC activation during sustained fear and change in behavioral avoidance was found (*P *> .05). The genotype-dependent correlation of ACC activation and within-session fear reduction could not be replicated (T-allele carriers: *r = *.126, *P = *.351; AA homozygotes: *r = *−.314, *P = *.135).

## Discussion

Given the relevance of the neuropeptide S system in fear processing, we investigated a possible association of the functional *NPSR1* rs324981 gene variant with neural correlates of sustained, phasic fear, and symptom reduction in a prototypical anxiety disorder not investigated yet. Regarding neural correlates of sustained and phasic fear, we partly replicated existing evidence. Further, we found a supracallosal ACC hypoactivation in T-allele carriers compared to AA homozygotes while processing phasic and sustained fear. However, only the latter could be replicated in a very small cluster in the replication sample. For the discovery sample, this effect was associated with within-session fear reduction during a VRET, but not with symptom reduction (pre to post) measured by the change of self-reported symptom change or avoidance behavior. Unexpectedly, we found no increased amygdala activation during phasic fear processing in *NPSR1* rs324981 T risk-allele carriers.

### Neural correlates of phasic and sustained fear condition

Although missing a healthy control group, our results indirectly represent evidence on the central role of amygdala processing phasic, but not sustained fear, in specific phobia (Münsterkotter et al. 2015, Brinkmann et al. 2017). It is in line with the idea that the amygdala is a central hub for bottom-up threat processing (Öhman 2005, Cisler and Koster 2010). In contrast, findings regarding a distinctive role of the insula in sustained and phasic fear processing are inconclusive. Whereas we found increased insula activation in the phasic compared to the sustained fear condition, other studies showed a relation between sustained fear and insula activation (Simmons et al. 2006, Wendt et al. 2008, Grupe et al. 2013, Münsterkotter et al. 2015). Still, most studies did not compare neural activation in sustained versus phasic fear condition. Thus, several studies also found phasic fear processing to be associated with increased insula activation (Straube et al. 2007, Mobbs et al. 2010, Herrmann et al. 2016).

Our results support previous evidence that the pregenual ACC is more involved in sustained than phasic fear processing (Straube et al. 2007, Alvarez et al. 2011, Grupe et al. 2013, Münsterkotter et al. 2015) and potentially displays a coping mechanism to deal with sustained hyperactivation in limbic areas like the amygdala. The pregenual ACC has been suggested to be involved in emotion regulation (Palomero-Gallagher et al. 2019) and in the top-down modulation of the amygdala recruited through the dorsal part of the ACC (Etkin et al. 2011). Pregenual parts of the ACC also have been associated with reward processing (Rolls 2019; Grabenhorst and Rolls, n.d.). Interestingly, in the phasic fear condition, we found replicable evidence for increased supracallosal ACC activation compared to the sustained condition. Supracallosal ACC, belonging to the dorsal part of ACC, activation has been associated with executive functions and processing of non-reward or punishers (Rolls et al. 2019), e.g. phobic stimuli or threat (Straube et al. 2007, Caseras et al. 2013). In the replication sample, we could confirm these results, emphasizing the robustness of our findings.

Activation in the BNST, often referenced as extended amygdala (Pedersen et al. 2019) which has been expected to be associated with the sustained fear condition, was not found to be increased. First of all, the BNST is a rather small region, which limits the likelihood of finding significant results. Another reason could be that in the paradigm only images of spiders, representing a more distal threat than moving or real spiders were used. Though only on a behavioral level, Grill and Haberkamp (Grill and Haberkamp 2023) showed that the movement of spiders is a relevant feature for individuals with spider phobia.

### Association between *NPSR1* genotype and neural correlates of phasic and sustained fear

The *NPSR1* rs324981 T risk-allele was associated with phasic and sustained fear processing, thus in both fear conditions T-allele carriers showed a significant hypoactivation in the supracallosal ACC compared to the AA homozygotes. The finding for the sustained fear condition could be confirmed by the replication sample, albeit in a substantially smaller cluster. Supracallosal ACC has been associated with the processing of unpleasant stimuli (Rolls 2019), which are of utmost importance in specific phobias. In line, Domschke et al. (2011) already showed decreased activity in the ACC in T-allele carriers suffering from panic disorder in response to fearful faces. Later, Domschke et al. (2017) also reported a reduction of fronto-limbic connectivity in healthy adolescent TT homozygotes, supporting the idea of an impaired top-down control as a result of hypoactivation in frontal areas such as the ACC. Further, there is supportive evidence in cell metabolism, that the functional *NPSR1* rs324981 gene variant indeed modulates ACC activity (Ruland et al. 2015). In healthy T-allele carriers, hyperactivation in frontal areas could be detected in an fNIRS study, but when associated with anxiety sensitivity, a negative correlation with prefrontal activation was found (Guhn et al. 2015). So, even subclinical anxiety could be driven by hypoactivation in frontal areas, as a form of decompensation in T-allele carriers. This refers back to the neural model of specific phobia as impaired regulation of limbic structures by prefrontal regions (Etkin et al. 2011), with the ACC as one hub.

To further disentangle the role of ACC hypoactivation for individual *NPSR1* rs324981 genotype prediction, we additionally conducted a machine learning analysis to predict individual *NPSR1* rs324981 genotype based on ACC activation during sustained fear processing (see Supplement 7). This analysis showed that activation patterns in the ACC during sustained fear processing are not suitable to predict individual *NPSR1* rs324981 genotype.

Against our expectations, we could not find a hyperactivation of the amygdala in T-allele carriers during the processing of phasic fear. Whereas increased amygdala activation in response to threatening stimuli could be shown in T-allele carriers with panic disorder (Gechter et al. 2019) and T-allele carriers without anxiety psychopathology (Dannlowski et al. 2011), other studies failed to replicate higher amygdala activity in T-allele carriers (Domschke et al. 2011, Siminski et al. 2021a). For our study, one possible explanation is a ceiling effect due to the high baseline activity of the amygdala in our sample only including individuals suffering from spider phobia (Böhnlein et al. 2021). For this reason and to differentiate between healthy and phobic T-allele carriers, a control group would be helpful in future studies. Regarding a potentially modulating effect of *NSPR1* rs324981 genotype on BNST activity during the processing of sustained fear, we could not replicate latest finding describing a differential involvement of the BNST in healthy T-allele carriers (Siminski et al. 2021a). This might be due to the conservative choice of a joint mask for all ROIs. We did not find any effect of *NPSR1* genotype on insula activity.

### Association with treatment-synchronized symptom change

We failed to discern an association between neural correlates of fear processing and symptom reduction. However, lower supracallosal ACC activity during sustained fear prior to treatment was associated with higher within-session fear reduction in T-allele carriers, but not in AA homozygotes. Of note, ACC hypoactivation of the T-allele carriers compared to healthy subjects could be an ACC hyperactivation after all. ACC pretreatment activation has been recently shown to be predictive for CBT-outcome in anxiety-related disorders (Lueken et al. 2016, Picó-Pérez et al. 2022) as well as across different therapies in affective disorders (Pizzagalli 2011), thus potentially marking a transdiagnostic marker of treatment response. Within-session fear reduction is discussed to be a valid predictor of treatment response (Rupp et al. 2017). In sum, the present results suggest that *NPSR1* rs324981 genotype modulates supracallosal ACC activation which indirectly affects within-session fear reduction as a putative core mechanism of exposure-based therapy.

### Limitations

Employing a replication sample in an imaging-genetic study allowed us to test the robustness of findings and represents a major strength of our study design. However, interpretation of our results demands the consideration of several limitations. The sample size is satisfying for fMRI studies, but rather small for a genetic investigation. Moreover, the sample consists only of participants suffering from specific phobia and limits generalizability. A healthy control group would have been helpful to further contextualize the findings, but was not targeted in the original investigation (Schwarzmeier et al. 2019). The resolution of the 3 T-MRI may not be sufficient for investigating a small structure like the BNST. In general, candidate gene approaches can be seen critically due to the higher risk of false-positive results, even though the association of *NPSR1* rs324981 genotype and panic disorder, subclinical anxiety, and fear has been demonstrated by several studies (Donner et al. 2010, Raczka et al. 2010, Dannlowski et al. 2011, Tupak et al. 2013, Domschke et al. 2017, Schiele et al. 2020). The fact that the association between *NPSR1* genotype and supracallosal ACC could be replicated strengthens the argument for a nonrandom effect. However, the fact, that the significant cluster in the replication sample was substantially smaller requires some reflection: Reasons could be lower strength or consistency of the signal in the replication sample, methodological differences as different scanners, or lower power in the replication sample, as the replication sample is smaller (*n* = 81 compared to *n* = 104 in the discovery sample). It is not possible to disentangle the reasons.

## Conclusion and perspective

Our study corroborates the importance of *NPSR1* gene variation in the etiopathophysiology of anxiety disorders. The hypothesis of fronto-limbic dysfunction resulting in an impaired top-down control may be central for future investigations. The contraintuitive negative association between ACC activation and within session fear reduction needs further investigation. As an outlook, the results suggesting an association of *NPSR1* rs324981 genotype with therapy-related parameters might eventually contribute to a more personalized treatment-response profile and hence inform personalized treatment approaches (Lueken et al. 2016).

## Supplementary Material

nsae054_Supp
