# Supplementary material for: Impact of NPSR1 gene variation on the neural correlates of phasic and sustained fear in spider phobia—an imaging genetics and independent replication approach
Source: Soc Cogn Affect Neurosci. 2024 Aug 21;19(1):nsae054. doi: 10.1093/scan/nsae054 (PMC11412251; doi:10.1093/scan/nsae054)
Supplement: nsae054_Supp [file nsae054_supp.zip › scan-23-253-File006.docx]

**Supplemental Material: *Impact of NPSR1 gene variation on the neural correlates of phasic fear and sustained anxiety in spider phobia – an imaging genetics and independent replication approach***

Supplement 1

Patients were excluded if they were diagnosed with another comorbid lifetime anxiety disorder (panic disorder, agoraphobia, social phobia, and generalized anxiety disorder). Additionally, any other psychiatric mental disorders except for a mild to moderate depression (not currently treated) or other specific phobias of the animal subtype (spider phobia had to be the primary diagnosis) were exclusion criteria. Neurological or systematic diseases, current or past psychotherapy unless for depression, pregnancy and current (psycho-) pharmacological treatment as well as pregnancy also led to an exclusion.

For N=200 patients (MS: n=113; WZ: n= 87) fMRI data, *NPSR1* rs324981 genotype extraction as well as treatment response data at post-assessment were available. N= 14 patients had to be excluded due to extensive movement (>3.3 mm; MS: n=6; WZ: n=5) or technical problems (MS: n=3), resulting in a final sample of N=104 in the discovery (MS) and N=81 in the replication sample (WZ).

Supplement 2

This sample overlaps with the sample used in Roesmann et al. (2023) except for N=17 in the MS and N=9 in the WZ sample, investigating the role of process variables during the exposure inrervention and is an extended sample (additional N=23 data sets from the MS site have been included and N=6 from the MS site and N=5 from the WZ site from the original sample have been excluded due to insufficient data quality) of the sample used in Leehr et al. (2021), investigating the predictive potential of clinical variables for treatment response. Recently, we published a paper including N=190 patients with spider phobia from the original study, with the focus on individual treatment response prediction based structural T1 and functional MRI data from the sustained and phasic fear paradigm (Chavanne *et al.*, 2023). The sample of the current studies differs in n=11 participants (n=3 patients from MS site included in the current study, but not in Chavanne et al. (2023) and n=8 patients (N=6 from MS site; N=2 from WZ site) not in included in the current study, but in Chavanne et al. (2023)).

Supplement 3

33 slices were used with a thickness of 3.8 mm (10% slice gap), a matrix of 64 x 64, resulting in a voxel size of 3.3 x 3.3 x 3.8 mm. The Field-of-view (FOV) was 210 mm. The echo time (TE) in Münster was 29 ms and in Würzburg 30 ms. The repetition time (TR) equaled 2 s and the flip angle (FA) was 90° at both locations. Slices covered the whole brain and were aligned transaxially parallel to the anterior-posterior commissural line with a tilted angle of 20°. To present the stimuli in Würzburg MR-compatible LCD goggles, whereas in Münster a back-projection monitor was used.

For all participants structural T1 images were visually checked for deviations, and we used the check homogeneity function of the CAT12 toolbox (https://neuro-jena.github.io/cat12-html//cat.html). Participants were excluded due to extensive movement (>3.3 mm; MS: n=6; WZ: n=5) or technical problems (MS: n=3) during the phasic and sustained fear paradigm.

Supplement 4

Table S1. Sample characteristics (mean and sd) for *NPSR1* rs324981 no risk (AA genotype) and risk-allele-carriers (AA/AT genotype) for the replication sample (WU).

|  |  | **AA** | **AT/TT** | **Test statistic** | **p-value** |
| --- | --- | --- | --- | --- | --- |
|  | N | 24 | 57 |  |  |
| *Sociodemographic variables* |  |  |  |  |  |
| Sex (f/m) | 81 | 20/4 | 50/7 | 0.277 | .599 |
| Age | 81 | 28.04 (9.26) | 28.89 (8.75) | -0.394 | .695 |
| Years of education | 81 | 13.54 (3.26) | 14.79 (3.28) | -1.566 | .121 |
|  |  |  |  |  |  |
| *Psychological Variables* |  |  |  |  |  |
| SPQ | 81 | 22.58 (2.26) | 23.51 (2.45) | -1.586 | .117 |
| BAT | 81 | 175.63 (50.36) | 166.35 (66.10) | 0.615 | .540 |
| FEAS Disgust | 80 | 109.21 (10.77) | 110.43 (12.71) | -0.411 | .682 |
| FEAS Fear | 80 | 99.00 (16.77) | 102.63 (12.87) | -1.052 | .296 |
| STAI-Trait | 81 | 37.00 (9.55) | 35.88 (9.11) | 0.499 | .619 |
| ASI-3 | 81 | 16.21 (11.39) | 14.72 (9.40) | 0.611 | .543 |
| UI-18 | 81 | 42.21 (18.05) | 39.37 (12.80) | 0.803 | .424 |
| BDI-II | 81 | 3.88 (4.48) | 3.05 (4.16) | 0.794 | .429 |
|  |  |  |  |  |  |
| *Intervention related variables* |  |  |  |  |  |
| Number of scenarios completed | 81 | 4.75 (0.74) | 4.65 (1.09) | 0.413 | .681 |
| VRET duration | 81 | 86.92 (28.40) | 85.44 (24.99) | 0.233 | .816 |
| SPQ change pre to post (in %) | 81 | 30.04 (18.01) | 35.60 (15.26) | -1.418 | .160 |
| BAT change pre to post (in %) | 81 | 48.74 (21.37) | 55.92 (29.04) | -1.092 | .278 |
| Within-session fear reduction | 81 | 43.36 (21.36) | 48.15 (18.40) | -1.019 | .311 |

*Notes:* The variable test-statistic comprise a Chi²-test for group differences regarding the sex distribution and in all other cases T-values. *= value indicate the highest symptom severity; f= female; m= male; SPQ Spider Phobia Questionnaire (Range 0-31*); BAT= Behavioural Avoidance Test (0-300*); FEAS= Questionnaire Assessing Fear of Spiders (0-126*); STAI-Trait= State-Trait Anxiety Inventory (0-80*); ASI-3= Anxiety Sensitivity Index (0-72*); UI-18= Intolerance of Uncertainty (6-30*); BDI-II= Beck’s Depression Inventory-II (0-63*).

Supplement 5

Table S2. Comparison of sample characteristics (mean and sd) at both sites (main effect site) stratified for *NPSR1* rs324981 genotype

|  |  | **Münster** | | **Würzburg** | | **Main effect site** |  |
| --- | --- | --- | --- | --- | --- | --- | --- |
|  |  | **AA** | **AT/TT** | **AA** | **AT/TT** | **Test statistic** | **p-value** |
|  | N | 30 | 74 | 24 | 57 | 0.014 | .907 |
| *Sociodemographic variables* |  |  |  |  |  |  |  |
| Sex (f/m) | 185 | 25/5 | 66/8 | 20/4 | 50/7 | .047 | .828 |
| Age |  | 27.1 (9.71) | 27.43 (7.99) | 28.04 (9.26) | 28.89 (8.75) | 1.019 | .312 |
| Years of education |  | 14.69 (2.58) | 14.66 (2.86) | 13.54 (3.26) | 14.79 (3.28) | -.558 | .577 |
|  |  |  |  |  |  |  |  |
| *Psychological Variables* |  |  |  |  |  |  |  |
| SPQ |  | 22.44 (1.91) | 22.61 (2.02) | 22.58 (2.26) | 23.51 (2.45) | 2.092 | .038 |
| BAT |  | 168.47 (76.85) | 163.19 (66.85) | 175.63 (50.36) | 166.35 (66.10) | .447 | .655 |
| FEAS Disgust |  | 106.93 (15.56) | 110.43 (14.79) | 109.21 (10.77) | 110.43 (12.71) | .318 | .751 |
| FEAS Fear |  | 101.97 (13.20) | 101.60 (10.58) | 99.00 (16.77) | 102.63 (12.87) | -.088 | .930 |
| STAI-Trait |  | 33.55 (6.88) | 35 (7.99) | 37.00 (9.55) | 35.88 (9.11) | 1.299 | .196 |
| ASI-3 |  | 13.48 (8.96) | 14.84 (9.48) | 16.21 (11.39) | 14.72 (9.40) | .493 | .622 |
| UI-18 |  | 36.90 (10.59) | 38.59 (12.30) | 42.21 (18.05) | 39.37 (12.80) | 1.079 | .282 |
| BDI-II |  | 3.28 (3.84) | 3.12 (3.04) | 3.88 (4.48) | 3.05 (4.16) | .237 | .813 |
|  |  |  |  |  |  |  |  |
| *Intervention related variables* |  |  |  |  |  |  |  |
| Number of scenarios completed |  | 4.27 (1.28) | 4.42 (1.06) | 4.75 (0.74) | 4.65 (1.09) | 1.915 | .057 |
| VRET duration |  | 78.3 (29.10) | 76.84 (23.47) | 86.92 (28.40) | 85.44 (24.99) | 2.286 | .023 |
| SPQ change pre to post (in %) |  | 30.96 (14.62) | 31.37 (13.22) | 30.04 (18.01) | 35.60 (15.26) | 1.233 | .219 |
| BAT change pre to post (in %) |  | 53.91 (31.46) | 47.69 (33.38) | 48.74 (21.37) | 55.92 (29.04) | .956 | .340 |
| Within-session fear reduction |  | 51.95 (21.68) | 55.43 (18.08) | 43.36 (21.36) | 48.15 (18.40) | -2.702 | .008 |

*Notes:* The variable test-statistic comprise a Chi²-test for group differences regarding the sex and genotype distribution and in all other cases T-values. *= value indicate the highest symptom severity; f= female; m= male; SPQ Spider Phobia Questionnaire (Range 0-31*); BAT= Behavioural Avoidance Test (0-300*); FEAS= Questionnaire Assessing Fear of Spiders (0-126*); STAI-Trait= State-Trait Anxiety Inventory (0-80*); ASI-3= Anxiety sensitivity index (0-72*); UI-18= Intolerance of Uncertainty (6-30*); BDI-II= Beck’s Depression Inventory-II (0-63*)

Supplement 6

In addition to the ROI approach, we performed exploratory whole-brain analyses regarding the association between *NPSR1* rs324981 genotype and neural correlates of phasic and sustained fear.

*Phasic fear.* For the phasic fear condition, we did not find an effect of *NPSR1* rs324981 genotype or regarding treatment response or an interaction effect of these variables.

*Sustained fear.* Again, whole brain analyses revealed no main effect of *NPSR1* rs324981 genotype, or an interaction effect.

Supplement 7

*Machine learning pipeline.*

We used the PHOTON-AI toolbox (see <https://photon-ai.com> (Leenings *et al.*, 2021)) version 2.4, building on python v3.9. Following guidelines by Poldrack and colleagues (Poldrack *et al.*, 2020), we applied a nested cross-validation scheme: 10 inner validation loops were used to optimize hyperparameters and 10x10 outer validation loops were used in a repeated k-fold scheme to estimate model performance (with 10% of the samples as test set in every step). The repeated k-fold scheme was used to achieve reliable metrics, since the metrics varied considerably between the different outer validation loops. Model optimization was based on the balanced accuracy. The pipeline consisted of a simple imputer to account for random missingness of activation data in single indivdual’s 1^st^-level t-maps, a standard scaler to scale data to unity variance and remove the mean and a principal component analysis (PCA). As classifier, we used either a support vector machine or a random forest.

Hyperparameter optimization was performed based on grid search. The hyperparameters entailed the inclusion of an imbalanced data transformer (Synthetic Minority Oversampling Technique) to achieve balanced target frequency, the choice of classifier as well as several parameters of the classifiers: For the support vector machine, these were the parameter C (1e-6, 1e-4, 1e-2, 1, 1e2, 1e4, or 1e6) and the choice of kernel (either linear or radial basis function). For the random forest classifier, these were the number of maximum features (either the square root or the log²) and the minimum number of samples per leaf (either 1%, 10% or 20%). To test for statistical significance of the model, we repeated the analysis 1000 times with permuted labels.

*Machine learning results.*

Balanced accuracy did not differ significantly from chance (p= 0.125), with a mean balanced accuracy of 0.552 (sd= 0.127; range: 0.276 and 0.90, see Table S3).

Table S3. Results of the machine learning analysis with 10x10 outer validation loops.

| Fold No. | Accuracy | Precision | Balanced Accuracy | Sensitivity | Specificity | AUC | Classifier | SVC Kernel | RFC Max Features | RFC Min Samples Leaf | Transformers |
| --- | --- | --- | --- | --- | --- | --- | --- | --- | --- | --- | --- |
| 1 | 0.632 | 0.706 | 0.429 | 0.857 | 0.000 | 0.429 | SVC, C = 1 | linear | n.a. | n.a. | PCA |
| 2 | 0.632 | 0.667 | 0.462 | 0.923 | 0.000 | 0.462 | SVC, C = 1 | linear | n.a. | n.a. | PCA |
| 3 | 0.474 | 0.636 | 0.436 | 0.539 | 0.333 | 0.436 | SVC, C = 1 | rbf | n.a. | n.a. | PCA |
| 4 | 0.421 | 0.625 | 0.442 | 0.385 | 0.500 | 0.442 | SVC, C = 1 | rbf | n.a. | n.a. | PCA |
| 5 | 0.474 | 0.667 | 0.481 | 0.462 | 0.500 | 0.481 | SVC, C = 1 | linear | n.a. | n.a. | None |
| 6 | 0.833 | 0.917 | 0.823 | 0.846 | 0.800 | 0.823 | SVC, C = 1 | rbf | n.a. | n.a. | PCA |
| 7 | 0.722 | 0.722 | 0.500 | 1.000 | 0.000 | 0.500 | SVC, C = 1 | linear | n.a. | n.a. | PCA |
| 8 | 0.556 | 0.727 | 0.508 | 0.615 | 0.400 | 0.508 | SVC, C = 1 | rbf | n.a. | n.a. | PCA |
| 9 | 0.667 | 0.818 | 0.646 | 0.692 | 0.600 | 0.646 | SVC, C = 1 | rbf | n.a. | n.a. | PCA |
| 10 | 0.722 | 0.786 | 0.623 | 0.846 | 0.400 | 0.623 | SVC, C = 1 | linear | n.a. | n.a. | PCA |
| 11 | 0.632 | 0.733 | 0.493 | 0.786 | 0.200 | 0.493 | SVC, C = 1 | linear | n.a. | n.a. | PCA |
| 12 | 0.316 | 0.500 | 0.276 | 0.385 | 0.167 | 0.276 | Random Forest | n.a. | log2 | 0.2 | PCA |
| 13 | 0.579 | 0.727 | 0.558 | 0.615 | 0.500 | 0.558 | SVC, C = 0.0001 | linear | n.a. | n.a. | PCA |
| 14 | 0.421 | 0.583 | 0.353 | 0.539 | 0.167 | 0.353 | SVC, C = 1 | rbf | n.a. | n.a. | PCA |
| 15 | 0.632 | 0.714 | 0.551 | 0.769 | 0.333 | 0.551 | SVC, C = 1 | rbf | n.a. | n.a. | PCA |
| 16 | 0.556 | 0.727 | 0.508 | 0.615 | 0.400 | 0.508 | SVC, C = 1 | rbf | n.a. | n.a. | PCA |
| 17 | 0.611 | 0.800 | 0.608 | 0.615 | 0.600 | 0.608 | SVC, C = 1 | rbf | n.a. | n.a. | PCA |
| 18 | 0.556 | 0.727 | 0.508 | 0.615 | 0.400 | 0.508 | SVC, C = 1 | linear | n.a. | n.a. | None |
| 19 | 0.611 | 0.714 | 0.485 | 0.769 | 0.200 | 0.485 | SVC, C = 0.0001 | linear | n.a. | n.a. | PCA |
| 20 | 0.778 | 0.800 | 0.662 | 0.923 | 0.400 | 0.662 | SVC, C = 1 | linear | n.a. | n.a. | PCA |
| 21 | 0.737 | 0.765 | 0.564 | 0.929 | 0.200 | 0.564 | SVC, C = 1 | linear | n.a. | n.a. | PCA |
| 22 | 0.579 | 0.667 | 0.468 | 0.769 | 0.167 | 0.468 | SVC, C = 1 | linear | n.a. | n.a. | PCA |
| 23 | 0.632 | 0.714 | 0.551 | 0.769 | 0.333 | 0.551 | SVC, C = 1 | rbf | n.a. | n.a. | PCA |
| 24 | 0.684 | 0.706 | 0.545 | 0.923 | 0.167 | 0.545 | SVC, C = 1 | linear | n.a. | n.a. | PCA |
| 25 | 0.790 | 0.800 | 0.712 | 0.923 | 0.500 | 0.712 | SVC, C = 1 | linear | n.a. | n.a. | PCA |
| 26 | 0.611 | 0.875 | 0.669 | 0.539 | 0.800 | 0.669 | SVC, C = 0.0001 | linear | n.a. | n.a. | PCA |
| 27 | 0.778 | 0.800 | 0.662 | 0.923 | 0.400 | 0.662 | SVC, C = 1 | rbf | n.a. | n.a. | None |
| 28 | 0.722 | 0.900 | 0.746 | 0.692 | 0.800 | 0.746 | SVC, C = 1 | rbf | n.a. | n.a. | PCA |
| 29 | 0.444 | 0.636 | 0.369 | 0.539 | 0.200 | 0.369 | SVC, C = 0.0001 | linear | n.a. | n.a. | PCA |
| 30 | 0.556 | 0.727 | 0.508 | 0.615 | 0.400 | 0.508 | SVC, C = 1 | linear | n.a. | n.a. | None |
| 31 | 0.684 | 0.833 | 0.657 | 0.714 | 0.600 | 0.657 | SVC, C = 1 | rbf | n.a. | n.a. | PCA |
| 32 | 0.632 | 0.875 | 0.686 | 0.539 | 0.833 | 0.686 | SVC, C = 1 | rbf | n.a. | n.a. | PCA |
| 33 | 0.526 | 0.700 | 0.519 | 0.539 | 0.500 | 0.519 | SVC, C = 1 | rbf | n.a. | n.a. | PCA |
| 34 | 0.632 | 0.667 | 0.462 | 0.923 | 0.000 | 0.462 | SVC, C = 1 | linear | n.a. | n.a. | PCA |
| 35 | 0.316 | 0.500 | 0.276 | 0.385 | 0.167 | 0.276 | SVC, C = 0.0001 | linear | n.a. | n.a. | PCA |
| 36 | 0.778 | 0.909 | 0.785 | 0.769 | 0.800 | 0.785 | SVC, C = 0.0001 | linear | n.a. | n.a. | PCA |
| 37 | 0.611 | 0.714 | 0.485 | 0.769 | 0.200 | 0.485 | SVC, C = 0.0001 | linear | n.a. | n.a. | PCA |
| 38 | 0.556 | 0.692 | 0.446 | 0.692 | 0.200 | 0.446 | SVC, C = 1 | linear | n.a. | n.a. | PCA |
| 39 | 0.500 | 0.750 | 0.531 | 0.462 | 0.600 | 0.531 | SVC, C = 0.0001 | linear | n.a. | n.a. | PCA |
| 40 | 0.778 | 0.909 | 0.785 | 0.769 | 0.800 | 0.785 | SVC, C = 1 | linear | n.a. | n.a. | PCA |
| 41 | 0.526 | 0.667 | 0.357 | 0.714 | 0.000 | 0.357 | SVC, C = 1 | rbf | n.a. | n.a. | PCA |
| 42 | 0.737 | 0.786 | 0.673 | 0.846 | 0.500 | 0.673 | SVC, C = 1 | linear | n.a. | n.a. | PCA |
| 43 | 0.579 | 0.857 | 0.647 | 0.462 | 0.833 | 0.647 | SVC, C = 0.0001 | linear | n.a. | n.a. | PCA |
| 44 | 0.421 | 0.625 | 0.442 | 0.385 | 0.500 | 0.442 | SVC, C = 1 | rbf | n.a. | n.a. | PCA |
| 45 | 0.632 | 0.688 | 0.506 | 0.846 | 0.167 | 0.506 | SVC, C = 0.0001 | linear | n.a. | n.a. | PCA |
| 46 | 0.667 | 0.818 | 0.646 | 0.692 | 0.600 | 0.646 | SVC, C = 1 | rbf | n.a. | n.a. | None |
| 47 | 0.778 | 0.765 | 0.600 | 1.000 | 0.200 | 0.600 | SVC, C = 1 | linear | n.a. | n.a. | PCA |
| 48 | 0.444 | 0.636 | 0.369 | 0.539 | 0.200 | 0.369 | SVC, C = 1 | linear | n.a. | n.a. | PCA |
| 49 | 0.778 | 0.765 | 0.600 | 1.000 | 0.200 | 0.600 | SVC, C = 1 | linear | n.a. | n.a. | PCA |
| 50 | 0.667 | 0.769 | 0.585 | 0.769 | 0.400 | 0.585 | SVC, C = 1 | linear | n.a. | n.a. | PCA |
| 51* | 0.947 | 0.933 | 0.900 | 1.000 | 0.800 | 0.900 | SVC, C = 0.0001 | linear | n.a. | n.a. | PCA |
| 52 | 0.579 | 0.778 | 0.603 | 0.539 | 0.667 | 0.603 | SVC, C = 1 | linear | n.a. | n.a. | PCA |
| 53 | 0.474 | 0.667 | 0.481 | 0.462 | 0.500 | 0.481 | SVC, C = 1 | rbf | n.a. | n.a. | PCA |
| 54 | 0.579 | 0.692 | 0.513 | 0.692 | 0.333 | 0.513 | SVC, C = 0.0001 | linear | n.a. | n.a. | PCA |
| 55 | 0.632 | 0.688 | 0.506 | 0.846 | 0.167 | 0.506 | SVC, C = 1 | rbf | n.a. | n.a. | None |
| 56 | 0.667 | 0.818 | 0.646 | 0.692 | 0.600 | 0.646 | SVC, C = 1 | rbf | n.a. | n.a. | PCA |
| 57 | 0.722 | 0.786 | 0.623 | 0.846 | 0.400 | 0.623 | SVC, C = 1 | rbf | n.a. | n.a. | PCA |
| 58 | 0.611 | 0.688 | 0.423 | 0.846 | 0.000 | 0.423 | SVC, C = 1 | linear | n.a. | n.a. | PCA |
| 59 | 0.722 | 0.786 | 0.623 | 0.846 | 0.400 | 0.623 | SVC, C = 1 | linear | n.a. | n.a. | PCA |
| 60 | 0.444 | 0.714 | 0.492 | 0.385 | 0.600 | 0.492 | SVC, C = 0.0001 | linear | n.a. | n.a. | PCA |
| 61 | 0.526 | 0.778 | 0.550 | 0.500 | 0.600 | 0.550 | SVC, C = 0.0001 | linear | n.a. | n.a. | PCA |
| 62 | 0.684 | 0.706 | 0.545 | 0.923 | 0.167 | 0.545 | SVC, C = 1 | linear | n.a. | n.a. | PCA |
| 63 | 0.684 | 0.706 | 0.545 | 0.923 | 0.167 | 0.545 | SVC, C = 0.0001 | linear | n.a. | n.a. | PCA |
| 64 | 0.526 | 0.700 | 0.519 | 0.539 | 0.500 | 0.519 | SVC, C = 1 | rbf | n.a. | n.a. | PCA |
| 65 | 0.632 | 0.688 | 0.506 | 0.846 | 0.167 | 0.506 | SVC, C = 1 | linear | n.a. | n.a. | PCA |
| 66 | 0.667 | 0.769 | 0.585 | 0.769 | 0.400 | 0.585 | SVC, C = 1 | linear | n.a. | n.a. | PCA |
| 67 | 0.611 | 0.750 | 0.546 | 0.692 | 0.400 | 0.546 | SVC, C = 1 | linear | n.a. | n.a. | PCA |
| 68 | 0.667 | 0.769 | 0.585 | 0.769 | 0.400 | 0.585 | SVC, C = 1 | rbf | n.a. | n.a. | PCA |
| 69 | 0.500 | 0.700 | 0.469 | 0.539 | 0.400 | 0.469 | SVC, C = 1 | linear | n.a. | n.a. | PCA |
| 70 | 0.722 | 0.833 | 0.685 | 0.769 | 0.600 | 0.685 | SVC, C = 0.0001 | linear | n.a. | n.a. | PCA |
| 71 | 0.526 | 0.692 | 0.421 | 0.643 | 0.200 | 0.421 | SVC, C = 1 | rbf | n.a. | n.a. | None |
| 72 | 0.421 | 0.667 | 0.487 | 0.308 | 0.667 | 0.487 | SVC, C = 0.0001 | linear | n.a. | n.a. | PCA |
| 73 | 0.842 | 0.857 | 0.795 | 0.923 | 0.667 | 0.795 | SVC, C = 0.0001 | linear | n.a. | n.a. | PCA |
| 74 | 0.790 | 0.800 | 0.712 | 0.923 | 0.500 | 0.712 | SVC, C = 1 | linear | n.a. | n.a. | PCA |
| 75 | 0.579 | 0.667 | 0.468 | 0.769 | 0.167 | 0.468 | SVC, C = 1 | rbf | n.a. | n.a. | None |
| 76 | 0.667 | 0.818 | 0.646 | 0.692 | 0.600 | 0.646 | SVC, C = 1 | linear | n.a. | n.a. | PCA |
| 77 | 0.778 | 0.846 | 0.723 | 0.846 | 0.600 | 0.723 | SVC, C = 0.0001 | linear | n.a. | n.a. | PCA |
| 78 | 0.444 | 0.667 | 0.431 | 0.462 | 0.400 | 0.431 | SVC, C = 1 | linear | n.a. | n.a. | None |
| 79 | 0.722 | 0.750 | 0.562 | 0.923 | 0.200 | 0.562 | SVC, C = 1 | linear | n.a. | n.a. | PCA |
| 80 | 0.500 | 0.667 | 0.408 | 0.615 | 0.200 | 0.408 | SVC, C = 0.0001 | linear | n.a. | n.a. | PCA |
| 81 | 0.790 | 0.917 | 0.793 | 0.786 | 0.800 | 0.793 | SVC, C = 1 | linear | n.a. | n.a. | PCA |
| 82 | 0.368 | 0.571 | 0.404 | 0.308 | 0.500 | 0.404 | SVC, C = 1 | linear | n.a. | n.a. | PCA |
| 83 | 0.684 | 0.769 | 0.635 | 0.769 | 0.500 | 0.635 | SVC, C = 1 | rbf | n.a. | n.a. | None |
| 84 | 0.579 | 0.647 | 0.423 | 0.846 | 0.000 | 0.423 | SVC, C = 1 | linear | n.a. | n.a. | PCA |
| 85 | 0.632 | 0.688 | 0.506 | 0.846 | 0.167 | 0.506 | SVC, C = 1 | rbf | n.a. | n.a. | None |
| 86 | 0.444 | 0.615 | 0.308 | 0.615 | 0.000 | 0.308 | SVC, C = 1 | rbf | n.a. | n.a. | PCA |
| 87 | 0.667 | 0.733 | 0.523 | 0.846 | 0.200 | 0.523 | SVC, C = 1 | linear | n.a. | n.a. | PCA |
| 88 | 0.611 | 0.875 | 0.669 | 0.539 | 0.800 | 0.669 | SVC, C = 1 | rbf | n.a. | n.a. | PCA |
| 89 | 0.778 | 0.846 | 0.723 | 0.846 | 0.600 | 0.723 | SVC, C = 1 | rbf | n.a. | n.a. | PCA |
| 90 | 0.722 | 0.750 | 0.562 | 0.923 | 0.200 | 0.562 | SVC, C = 0.0001 | linear | n.a. | n.a. | PCA |
| 91 | 0.632 | 0.733 | 0.493 | 0.786 | 0.200 | 0.493 | SVC, C = 0.0001 | linear | n.a. | n.a. | PCA |
| 92 | 0.684 | 0.769 | 0.635 | 0.769 | 0.500 | 0.635 | SVC, C = 1 | linear | n.a. | n.a. | PCA |
| 93 | 0.790 | 0.846 | 0.756 | 0.846 | 0.667 | 0.756 | SVC, C = 0.0001 | linear | n.a. | n.a. | PCA |
| 94 | 0.421 | 0.625 | 0.442 | 0.385 | 0.500 | 0.442 | SVC, C = 1 | rbf | n.a. | n.a. | PCA |
| 95 | 0.421 | 0.625 | 0.442 | 0.385 | 0.500 | 0.442 | SVC, C = 1 | rbf | n.a. | n.a. | PCA |
| 96 | 0.722 | 0.833 | 0.685 | 0.769 | 0.600 | 0.685 | SVC, C = 0.0001 | linear | n.a. | n.a. | PCA |
| 97 | 0.833 | 0.857 | 0.762 | 0.923 | 0.600 | 0.762 | SVC, C = 0.0001 | linear | n.a. | n.a. | PCA |
| 98 | 0.500 | 0.643 | 0.346 | 0.692 | 0.000 | 0.346 | SVC, C = 0.0001 | linear | n.a. | n.a. | PCA |
| 99 | 0.500 | 0.643 | 0.346 | 0.692 | 0.000 | 0.346 | SVC, C = 1 | rbf | n.a. | n.a. | PCA |
| 100 | 0.611 | 0.750 | 0.546 | 0.692 | 0.400 | 0.546 | SVC, C = 0.0001 | linear | n.a. | n.a. | PCA |

*Notes:* AUC= Area under the Curve; SVC= Support vector classification; RFC= Random Forest Classifier; PCA= Prinicipal component analysis; n.a.= not applicable.

References:

Chavanne, A. V., Meinke, C., Langhammer, T., et al. (2023). Individual-Level Prediction of Exposure Therapy Outcome Using Structural and Functional MRI Data in Spider Phobia: A Machine-Learning Study C. Carona (ed). *Depression and Anxiety*, **2023**, 1–11

Leehr, E.J., Roesmann, K., Böhnlein, J., et al. (2021). Clinical predictors of treatment response towards exposure therapy in virtuo in spider phobia: A machine learning and external cross-validation approach. *Journal of anxiety disorders*, **83**

Leenings, R., Winter, N.R., Plagwitz, L., et al. (2021). PHOTONAI-A Python API for rapid machine learning model development. *PloS one*, **16**

Poldrack, R.A., Huckins, G., Varoquaux, G. (2020). Establishment of Best Practices for Evidence for Prediction: A Review. *JAMA psychiatry*, **77**, 534–40

Roesmann, K., Leehr, E.J., Böhnlein, J., et al. (2023). Mechanisms of action underlying virtual reality exposure treatment in spider phobia: Pivotal role of within-session fear reduction. *Journal of anxiety disorders*, **100**
